# A randomized trial of grant writing coaching groups: Baseline analysis of early-career scientists’ research background, demographics, and mentorship variables

**DOI:** 10.1371/journal.pone.0334039

**Published:** 2025-10-22

**Authors:** Anne Marie Weber-Main, Richard McGee, Melanie Steiner, Jeffrey Engler, Harlan P. Jones, Jessica M. Faupel-Badger, Alperen Korkmaz, Andrew K. Langi, Patrick O. Monahan, Kolawole S. Okuyemi

**Affiliations:** 1 Department of Medicine, University of Minnesota Medical School, Minneapolis, Minnesota, United States of America; 2 Faculty Affairs, Northwestern University Feinberg School of Medicine, Chicago, Illinois, United States of America; 3 Department of Family Medicine, Indiana University School of Medicine, Indianapolis, Indiana, United States of America; 4 Office of Research, Dean’s Office, University of California Davis School of Medicine, Sacramento, California, United States of America; 5 Department of Microbiology, Immunology, and Genetics, College of Biomedical and Translational Sciences, University of North Texas Health, Fort Worth, Texas, United States of America; 6 Institute for Health Disparities, University of North Texas Health, Fort Worth, Texas, United States of America; 7 National Cancer Institute, National Institutes of Health, Rockville, Maryland, United States of America; 8 Department of Biostatistics and Health Data Science, Indiana University School of Medicine, Indianapolis, Indiana, United States of America; University of North Carolina at Greensboro, United States of America

## Abstract

**Introduction:**

Racial, ethnic, and gender disparities in academic career advancement persist in biomedical disciplines. One approach to addressing this problem is systematizing access to mentorship in critical skills such as grant writing. This report summarizes the baseline characteristics of early-career investigators who enrolled in a randomized trial of a group coaching intervention focused on National Institutes of Health (NIH) grant application development.

**Methods:**

Surveys assessed participants’ demographic characteristics, research focus, prior publications and grant submissions, self-efficacy for grantsmanship and career advancement, and access to mentorship. Two-sided t-test and Fisher’s exact test were performed to compare baseline variables by gender identity (male/female) and by background from a racial or ethnic population that is an underrepresented minority group in biomedical research (non-URM/URM).

**Results:**

The study sample includes 271 faculty and 96 postdoctoral fellows. Sixty-two percent of faculty and 76.0% of postdoctoral fellows identified as female. Nearly half (45.4% of faculty, 49.0% of postdocs) were from URM populations in biomedical research. At baseline, most were conducting clinical and translational research at institutions with high levels of research activity. Past submission of NIH R-series applications was limited; 29.9% of faculty had submitted K applications. On average, participants had moderate levels of self-efficacy (in grantsmanship and career advancement) and research-related mentoring support. Male and non-URM participants had a higher mean number of previous publications. For the remaining variables, there were no or minimal differences by gender identity and URM status.

**Conclusions:**

Early-career investigators from diverse backgrounds are motivated to engage in external grant writing coaching programs regardless of existing mentorship and other supports at their home institutions, suggesting that grant coaching can provide complementary value.

## Introduction

Racial, ethnic, and gender disparities continue to persist in the advancement of individuals within biomedical careers, despite decades of efforts to mitigate this phenomenon [1–5]. One significant and well-documented disparity has been in award rates for grants from the National Institutes of Health (NIH), with lower rates observed for members of populations that are underrepresented in biomedical research [1,6–10]. The NIH Diversity Program Consortium (DPC) was launched in 2014 to help address these disparities and support greater workforce diversification [11,12]. The DPC has directed many of its efforts at improving the quality of, and increasing access to, mentoring and professional development programming tailored to individuals at different training and career levels [13]. At the postdoctoral and faculty levels, a core element of these efforts has been to provide national, structured group coaching models for career development, with an emphasis on support for research application preparation that supplements local mentoring resources [14–21].

In 2019, our research team received a 5-year U01 award as part of the DPC’s National Research Mentoring Network (NRMN) initiative [22]. In this project [14], we are conducting a group-randomized trial to test variations of a novel group-based coaching intervention for early-career biomedical researchers (faculty and postdoctoral fellows) who are developing an NIH K- or R-series application. Additionally, we aim to identify contextual factors—those associated with individual participants, their engagement with the intervention, and their institutional setting, including the local mentoring context [22]—that might influence the intervention’s impact on application submission and funding rates. Our core intervention is grounded in an intensive group coaching model [14,15,20]. Participants are trained in the rhetorical patterns common to NIH applications and supported during several months of active application writing. They receive feedback and psychosocial support from trained coaches (senior faculty members) and peers (other coaching group participants) on a biweekly basis. Study arms are distinguished by different durations of coaching and different levels of engagement of the participant’s self-selected scientific advisor with the group coaching process. We developed the project’s coaching approaches and other programmatic features by drawing on our previous experience delivering a variety of research and grant writing training programs as part of the NRMN Professional Development Core during the first 5 years of the DPC [14,15,17,20,23–26].

When assessing the effectiveness of grant writing interventions such as ours, it is important to consider participants’ background characteristics. Factors such as scientific discipline, employment level, prior grants, publication record, and access to resources and mentoring support could have confounding effects on our study’s outcomes, and these factors may be affected by investigators’ gender, race, and ethnicity. For example, Shavers et al (2005) found that minority investigators reported a lack of mentoring and institutional support, along with social, cultural, and environmental factors, as obstacles to success [27]. Haggerty and Fenton (2018) studied outcomes of first-time NIH grantees and found that factors such as number of applications submitted per year, submission of renewal applications, and submission of applications to multiple NIH Institutes were associated with future funding [28]. Another study that followed the trajectory of recipients of K99/R00 awards found that men and those at well-funded institutions had greater subsequent funding success [29]. Other factors that influence funding rates include topic choice [10] and the NIH Institute or Center to which specific topics are assigned [30]. Given these documented inequities, a concern when starting our study was whether we could enroll and subsequently randomize a diverse sample in which investigators from gender and racial and ethnic populations that are underrepresented in biomedical research would have similar baseline characteristics as those from majority groups. (Racial and ethnic groups underrepresented in biomedical research include individuals who identify fully or in part as Black and African American, Hispanic or Latino, American Indian or Alaska Native, or Native Hawaiian and Other Pacific Islander [31].) Without reasonable equivalency in participants’ research backgrounds, any observed differences in outcomes by gender and racial and ethnic identity could be due to different starting points rather than differential benefits of the intervention.

This article provides a summary of our study participants’ demographic characteristics, home institution, research focus, publication record, history of prior grant application submission and funding, self-efficacy in grantsmanship and career advancement, and engagement with mentors at the time of study enrollment. These baseline variables will be used in planned future analyses to determine if they predict the primary outcomes of grant application submission and funding rates. Other measures were collected to assess change over time (post intervention and follow-up) and/or as common metrics to enable comparisons with other U01 studies funded as part of the NRMN consortium. Taken together, these data describe the characteristics of early-career investigators who were motivated to engage in extended grant writing coaching offered outside of their institutions.

## Methods

### Overview of study protocol

The study received an exemption determination by the Institutional Review Board (IRB) at the University of Utah (#00113440). Recruitment began October 01, 2019, and ended June 30, 2022. The participant consent process was administered electronically using the secure web-based data capture platform REDCap (Research Electronic Data Capture) [32]. The IRB waived documentation of signed consent.

A complete description of our study protocol has been published [14]. In brief, early-career investigators were invited through NRMN announcements to apply for participation in a pragmatic, group-randomized trial to test the effectiveness of variations of a group coaching intervention focused on NIH grant application preparation. Eligible to participate were faculty or postdoctoral fellows who were actively developing a new or revised NIH application (Rs or Ks); had never been PI of an R01 grant; appeared to have sufficient research training, prior publications, and preliminary data to support their targeted funding mechanism; were employed at an institution with the scientific and administrative resources needed to support the grant; and had adequate time to commit to full participation in the study. We intentionally oversampled men and participants from racial and ethnic underrepresented minority (URM) groups relative to the pool of applicants to increase their proportion within the study sample and allow for comparisons among demographic groups.

Our study’s enrollment target was met through the engagement of 6 distinct cohorts, initiated every 6 months from January 2020 through June 2022. Participants were enrolled as dyads with a self‑selected scientific advisor in their research area, then placed into coaching groups of 4–5 dyads each. Entire coaching groups were randomized to 1 of 4 study arms that differed on 2 factors: duration of coaching support (5 months of group coaching only, versus 5 months of group coaching plus access to an additional 18 months of one‑on‑one coaching); and mode of engaging scientific advisors with the group coaching process (structured versus unstructured engagement). Coaches were established NIH-funded investigators trained in the intervention’s coaching approaches described previously [20].

The study’s primary outcome variable is participants’ success in acquiring external research grants, predominantly from the NIH, at 30 months after the initiation of coaching. Secondary outcomes include application submission rates, whether the application was discussed by the study section, application score after review, and resubmission rates. Qualitative data from semi-structured interviews with participants and coaches will undergo thematic and directed content analysis to ascertain contextual factors (individual, group, or institutional) that may have influenced these outcomes. Additionally, we are examining pre- and post-intervention changes in a set of common measures collected across other NRMN U01 projects, including participants’ perceptions of their self-efficacy for grant application development, self-efficacy for career advancement, and receipt of resources and mentorship at their current institution.

Quantitative and qualitative findings on the impact of the study’s coaching interventions are being analyzed and will be presented in future manuscripts. This article reports solely on the pre-intervention (baseline) characteristics of the study’s participants, as outlined next.

### Baseline variables and data collection

Participants’ baseline characteristics were drawn from their responses to 2 instruments: the application to join the study (used to determine eligibility) and the baseline survey completed at enrollment. Both surveys were administered using REDCap.

We collected data for the demographic variables of age, gender identity, race and ethnicity, education, training/career stage, and current institution. Participants’ research backgrounds were assessed via items that queried the type of research they were engaged in, number of peer-reviewed publications they had authored, type of grant applications they had previously submitted, and whether these applications had been funded.

In addition to data on participants’ demographics and prior research experiences, we assessed at baseline their perceived confidence in developing research grant applications, self-efficacy for advancing in their careers, and access to mentorship. Specifically, our baseline survey included the following measures: the Grantsmanship Self-Efficacy Scale, a validated 19-item scale derived from the Clinical Research Assessment Inventory to measure self-efficacy in conceptualizing, designing, and obtaining funding for a research project [26,33]; two validated scales developed for use in the faculty C-Change study—the 3-item Self-Efficacy for Career Advancement Scale [34,35] and the 6-item Key Components of Mentoring Scale [36]; and a new measure created by the NRMN’s Coordinating Center to elucidate respondents’ perspectives on how frequently they discussed specific professional development topics within their current mentoring relationships.

### Statistical analyses

We calculated descriptive statistics, including mean and standard deviation, for each continuous variable and the frequency, percentage, and composite scores for each categorical variable. Scales were measured using items reported on an anchored Likert-type metric. We conducted t-tests and Fisher’s exact tests as applicable to compare differences in select variables by gender identity (female versus male) and by whether one’s racial and ethnic background corresponded to an underrepresented minority population in biomedical research (URM versus non-URM). We did not adjust for multiple comparisons in the primary analyses of gender identity (female versus male) and underrepresented minority status (URM versus non-URM) on grant application submission and funding by grant mechanism, because these comparisons were pre-planned, and because small samples in several cells made Type II error as important as Type I error. However, we did conduct a Bonferroni adjustment to the comparison of topics discussed with mentors. All statistical tests were 2-sided. Analyses were conducted in RStudio.

## Results

### Demographic characteristics and research background of study participants

Our sample of 367 individuals included a mix of faculty (271, 73.8%) and postdoctoral fellows (96, 26.2%). Their demographic characteristics are summarized in Table 1. Mean age was 41.1 years for faculty and 36.8 years for postdoctoral fellows. Approximately two-thirds (65.9%) of the total sample were female. Almost half of the participants self-identified as white non-Hispanic (28.1%) or Asian (21.8%). The remainder self-identified as being from a racial or ethnic group that is underrepresented in biomedical research, including Black or African American (24.0%), Hispanic or Latino (14.7%), Native Hawaiian or Other Pacific Islander (less than 1%), American Indian or Alaska Native (less than 1%), or from more than 1 racial or ethnic URM group (6.5%).

**Table 1:** Demographic Characteristics, Research Focus, and Publication Record of Study Participants.

	Total Sample	Faculty	Postdoctoral Fellows
Variable^a^	(N = 367)	(N = 271)	(N = 96)
**Age in Years, mean (SD)**	39.9 (6.3)	41.1 (6.0)	36.8 (6.0)
**Gender Identity, n (%)**			
Male	120 (32.7)	98 (36.2)	22 (22.9)
Female	242 (65.9)	169 (62.4)	73 (76.0)
Other	5 (1.4)	4 (1.5)	1 (1.0)
**Race and Ethnicity** ^**b**^ **, n (%)**			
American Indian or Alaska Native	1 (0.3)	1 (0.4)	0 (0.0)
Asian	80 (21.8)	64 (23.6)	16 (16.7)
Black or African American	88 (24.0)	65 (24.0)	23 (24.0)
Hispanic or Latino	54 (14.7)	37 (13.7)	17 (17.7)
Native Hawaiian or Other Pacific Islander	3 (0.8)	3 (1.1)	0 (0.0)
White	103 (28.1)	75 (27.7)	28 (29.2)
Other	9 (2.5)	5 (1.8)	4 (4.2)
More than one race	29 (7.9)	21 (7.7)	8 (8.3)
**URM** ^**c**^ **, n (%)**			
Non-URM	197 (53.7)	148 (54.6)	49 (51.0)
URM	170 (46.3)	123 (45.4)	47 (49.0)
**Highest Degree Completed, n (%)**			
PhD	287 (78.2)	203 (74.9)	84 (87.5)
MD, DDS, DMD	42 (11.5)	38 (14.0)	4 (4.2)
MD/PhD, PharmD/PhD, DVM/PhD	28 (7.6)	20 (7.4)	8 (8.3)
Other Doctorate^**d**^	10 (2.7)	10 (3.7)	0 (0)
**Employment Levels n (%)**			
Postdoctoral Fellows	96 (26.2)	–	96 (26.2)
Instructor	16 (4.4)	16 (4.4)	–
Assistant Professor	221 (60.2)	221 (60.2)	–
Associate Professor	29 (7.9)	29 (7.9)	–
Professor	3 (0.8)	3 (0.8)	–
Adjunct Professor	2 (0.5)	2 (0.5)	–
**Carnegie Classification of Current Institution** ^**e**^ **, n (%)**			
Doctoral Very High Research Activity	233 (63.8)	161 (60.1)	72 (74.2)
Doctoral High Research Activity	39 (10.7)	37 (13.8)	2 (2.1)
Special Focus Four-Year	66 (18.1)	43 (16.0)	23 (23.7)
Doctoral/Professional Universities	14 (3.8)	14 (5.2)	0 (-)
Baccalaureate or Master’s Colleges and Universities	13 (3.6)	13 (4.9)	0 (-)
**Minority-Serving Institution** ^**e,f**^ **, n (%)**			
No	330 (90.4)	239 (89.2)	91 (93.8)
Yes	35 (9.6)	29 (10.8)	6 (6.2)
**Research Focus, n (%)**			
Laboratory Based	132 (36.0)	100 (36.9)	32 (33.3)
Social/Behavioral	115 (31.3)	84 (31.0)	31 (32.3)
Clinical	82 (22.3)	61 (22.5)	21 (21.9)
Epidemiology/Data Science	38 (10.4)	26 (9.6)	12 (12.5)
**Peer-reviewed Publications, mean (SD)**	19.6 (15.6)	22.5 (16.5)	11.2 (7.9)

^**a**^For categorical variables, data are presented as numbers with percentages (%) in parentheses. For continuous variables, data are presented as means with standard deviations (SD) in parentheses. ^**b**^Respondents were allowed to select multiple responses. ^**c**^The under-represented minority (URM) variable is derived based on race and ethnicity categories that include individuals who identified either exclusively or in combination as American Indian or Alaska Native, Black or African American, Hispanic or Latino, Native Hawaiian and Other Pacific Islander. Of 29 respondents with multiple race and ethnicity identities, 5 selected combinations not considered URM (e.g., White + Asian). ^**d**^Other doctoral degrees included DPT, DrPH, ScD, DSW, DEd, AuD, and PharmD. ^**e**^Institutional classification was known for 365 study participants. ^**f**^Minority-serving institutions are defined as institutions of higher education that serve minority populations, including Historically Black Colleges and Universities, Hispanic-Serving Institutions, Tribal Colleges and Universities, and Asian American and Pacific Islander Serving Institutions.

Data on participants’ educational backgrounds, current institution, type of research they conduct, and peer-reviewed publications are also provided in Table 1. Individuals with research-focused doctorates (PhD or combined PhD degrees, such as MD/PhD) were predominant in our sample (85.8%). A very high proportion of participants (>95%) were employed at institutions with a Carnegie classification indicative of high or very high research activities and resources. (Note that institutions classified as Special Focus Four-Year and Doctoral/Professional Universities largely include free-standing medical schools and research institutes; these are counted among high and very high research institutions for the purpose of this study.) Only a very small number of faculty and no postdoctoral fellows were employed at Baccalaureate or Master’s Colleges and Universities. Approximately 11% of faculty and 6% of postdoctoral fellows were employed at Minority-Serving Institutions.

Participants were pursuing diverse types of research. Thirty-six percent were focused on laboratory-based research, 31.3% on social/behavioral research, 22.3% on clinical research, and 10.4% on epidemiology/data science research. The mean number of peer-reviewed publications previously authored by participants was 22.5 for faculty and 11.2 for postdoctoral fellows. However, the standard deviation (SD) was large for both groups, indicating a wide range for this variable. On average, men had a higher mean number of prior publications than women (22.1 ± 14.3 vs 18.4 ± 16.1; p = 0.029), and non-URM participants had more than URM participants (22.7 ± 15.9 vs 15.9 ± 13.2; p = 0.0001).

At baseline, participants indicated the specific type of grant application they were planning to write during the group coaching intervention (Table 2). About half of the faculty intended to apply for an NIH Research Project Grant (R01)—a primary target outcome for the trial. Most of the remainder were working on applications for other NIH R-series or K-series (Career Development) grants. A large majority (81.3%) of postdoctoral fellows planned to apply for NIH Career Development grants (predominantly K01 and K99/R00). A small subset of our total sample (<4% of faculty and 4.2% of postdoctoral fellows) sought to develop applications for other extramural funding agencies that had R- or K-equivalent grant mechanisms using the same or similar application format and review criteria.

**Table 2:** Distribution of Targeted Application Submissions by Career Level.

	Faculty (N = 271)	Postdoctoral Fellow (N = 96)
Targeted Application	n (%)	n (%)
NIH R01	134 (49.4)	1 (1.0)
NIH R03, R15, R21, R33/34	65 (24.0)	12 (12.5)
NIH K01, K08, K22, K23, K99/R00	63 (23.3)	78 (81.3)
Other Federal	0 (-)	1 (1.0)
Other	9 (3.3)	4 (4.2)

NIH, National Institutes of Health.

### Baseline data on history of submitted applications and funded grants

Participants’ prior experiences in grant seeking for a variety of funders and grant mechanisms are synthesized in Tables 3 and 4 for faculty and postdoctoral fellows, respectively. These data are for individual participants, not applications, and each person is counted only once within each application category. Participants are included in the “Funded” group if they received at least 1 award of that application type, regardless of how many they submitted. Participants are included in the “Submitted, Not Funded” group if they submitted at least 1 application in that application category but had no funding success. The “No submission” group includes participants who did not report any applications for the specified grant category.

**Table 3:** Grant Application Submission and Funding History of Faculty Members By Gender Identity and URM Status.

		Gender Identity			URM Status		
Type of Application^a^	Faculty^b^ n (%)	Male n (%)	Female n (%)	p-values^c^	Non-URM n (%)	URM n (%)	p-values^c^
**Total**	267 (100)	98 (36.7)	169 (63.3)		145 (54.3)	122 (45.7)	
**NIH Fellowship (F31, F32, other F)**				0.628			0.172
Funded	25 (9.4)	7 (7.1)	18 (10.7)		9 (6.2)	16 (13.1)	
Submitted, Not Funded	23 (8.6)	8 (8.2)	15 (8.9)		13 (9.0)	10 (8.2)	
No submission	219 (82.0)	83 (84.7)	136 (80.5)		123 (84.8)	96 (78.7)	
**NIH K99/R00**				**0.012** ^**d**^			0.205
Funded	9 (3.4)	4 (4.1)	5 (3.0)		4 (2.8)	5 (4.1)	
Submitted, Not Funded	10 (3.7)	8 (8.2)	2 (1.2)		8 (5.5)	2 (1.6)	
No submission	248 (92.9)	86 (87.8)	162 (95.9)		133 (91.7)	115 (94.3)	
**NIH K-series & Other Federal Career Development** ^**e**^				0.089			0.467
Funded	53 (19.9)	13 (13.3)	40 (23.7)		25 (17.2)	28 (23.0)	
Submitted, Not Funded	27 (10.1)	9 (9.2)	18 (10.7)		16 (11.0)	11 (9.0)	
No submission	187 (70.0)	76 (77.6)	111 (65.7)		104 (71.7)	83 (68.0)	
**Non-federal Career Development Applications**				0.351			0.303
Funded	12 (4.5)	2 (2.0)	10 (5.9)		6 (4.1)	6 (4.9)	
Submitted, Not Funded	12 (4.5)	5 (5.1)	7 (4.1)		4 (2.8)	8 (6.6)	
No submission	243 (91.0)	91 (92.9)	152 (89.9)		135 (93.1)	108 (88.5)	
**NIH R-series and SC Applications** ^**f**^				0.183			0.217
Funded	12 (4.5)	2 (2.0)	10 (5.9)		8 (5.5)	4 (3.3)	
Submitted, Not Funded	90 (33.7)	38 (38.8)	52 (30.8)		54 (37.2)	36 (29.5)	
No submission	165 (61.8)	58 (59.2)	107 (63.3)		83 (57.2)	82 (67.2)	
**Other Extramural Applications** ^**g**^				0.149			0.579
Funded	126 (47.2)	45 (45.9)	81 (47.9)		70 (48.3)	56 (45.9)	
Submitted, Not Funded	51 (19.1)	21 (21.4)	30 (17.8)		30 (20.7)	21 (17.2)	
No submission	90 (33.7)	32 (32.7)	58 (34.3)		45 (31.0)	45 (36.9)	

^**a**^For each grant application type, participants were placed into 1 of 3 categories: Funded (received at least 1 grant of this type), Submitted, Not funded (applied for at least 1 grant of this type, but none were funded), or No submission (did not submit any applications of this type). ^**b**^Excludes 4 faculty members who did not identify as either female or male. ^**c**^P-value by Fisher’s exact test. ^**d**^P-value<0.05 indicates a significant difference. ^**e**^Includes K01, K08, K12, K22, K23, K24, KL2 applications and other federal applications comparable to the NIH K mechanism. ^**f**^Includes R01, R03, R15, R21, R33/35 applications. ^**g**^Includes applications to foundations, local and state agencies, federal agencies other than NIH, and NIH mechanisms not previously named (e.g., NIH pilot/feasibility grants, diversity supplements).

**Table 4:** Grant Application Submission and Funding History of Postdoctoral Fellows by Gender Identity and URM Status.

		Gender Identity			URM Status		
Type of Application^a^	Post-doctoral Fellow^b^ n (%)	Male n (%)	Female n (%)	p-values^c^	Non-URM n (%)	URM n (%)	p-values^c^
**Total**	95 (100)	22 (23.2)	73 (76.8)		49 (51.6)	46 (48.4)	
**NIH Fellowship (F31, F32, other F)**				0.847			0.943
Funded	11 (11.6)	2 (9.1)	9 (12.3)		5 (10.2)	6 (13)	
Submitted, Not Funded	13 (13.7)	2 (9.1)	11 (15.1)		7 (14.3)	6 (13)	
No submission	71 (74.7)	18 (81.8)	53 (72.6)		37 (75.5)	34 (73.9)	
**NIH K99/R00**				0.487			0.363
Funded	1 (1.1)	0 (0.0)	1 (1.4)		0 (0)	1 (2.2)	
Submitted, Not Funded	5 (5.3)	2 (9.1)	3 (4.1)		4 (8.2)	1 (2.2)	
No submission	89 (93.7)	20 (90.9)	69 (94.5)		45 (91.8)	44 (95.7)	
**Non-federal Career Development Applications**				0.999			0.479
Funded	2 (2.1)	0 (0)	2 (2.7)		0 (0)	2 (4.3)	
Submitted, Not Funded	2 (2.1)	0 (0)	2 (2.7)		1 (2)	1 (2.2)	
No submission	91 (95.8)	22 (100)	69 (94.5)		48 (98)	43 (93.5)	
**Other Extramural Applications** ^**d**^				0.228			0.776
Funded	21 (22.1)	3 (13.6)	18 (24.7)		10 (20.4)	11 (23.9)	
Submitted, Not Funded	6 (6.3)	0 (0.0)	6 (8.2)		4 (8.2)	2 (4.3)	
No submission	68 (71.6)	19 (86.4)	49 (67.1)		35 (71.4)	33 (71.7)	

^**a**^For each grant application type, participants were placed into 1 of 3 categories: Funded (received at least 1 grant of this type), Submitted, Not funded (applied for at least 1 grant of this type, but none were funded), or No submission (did not submit any applications of this type). ^**b**^Excludes 1 postdoctoral fellow who did not identify as either female or male. ^**c**^P-value by Fisher’s exact test. P-value<0.05 indicates a significant difference. ^**d**^Includes applications to foundations, local and state agencies, federal agencies other than NIH, and NIH mechanisms not previously named (e.g., NIH pilot/feasibility grants, diversity supplements).

Among faculty, 9.4% had obtained individual NIH fellowships during training. Close to 9% had submitted fellowship applications but not been funded. A small number of faculty had applied for and received K99/R00 awards (3.4%), whereas 24.4% had received other types of K awards or similar career development grants from either federal or foundation sources. Per the study’s eligibility criteria, no participants had ever received an R01 grant. However, 4.5% of faculty participants had received other types of R-series or SC research grants, and another 33.7% had submitted an application for an R-series grant (including R01) but not been funded. Lastly, 66.3% of faculty had submitted some other type of application for extramural funding (a mixture of federal, foundation, state, or local grants), and 71.2% of these individuals were successful in being awarded at least 1 such grant.

No statistically significant differences were observed in either submissions or awards by URM status for faculty. Just 1 significant gender difference in funding and submission outcomes was observed, and this was for NIH K99/R00 applications (Table 3). Post hoc pairwise analyses were performed, which indicated significant differences between the “Submitted but Not Funded” and “No Submission” categories (adjusted p = 0.017). Specifically, men were more likely than women to submit a K99/R00 without receiving funding, whereas women were more likely than men to fall into the “No Submission” category. However, the total number of K99/R00 submissions in the sample was small, which may limit the practical value of these observed statistical differences.

Among postdoctoral fellows, 11.6% had obtained individual NIH fellowships, and another 13.7% had submitted fellowship applications but not been funded. A small number of postdoctoral fellows had applied for and been funded by K99/R00 awards (1.1%) and other types of career development grants (2.1%) from non-federal sources. Lastly, 22.1% had been awarded at least 1 other type of extramural grant. Among postdoctoral fellows, no significant differences were observed in either submissions and awards by gender identity or URM status.

### Grantsmanship self-efficacy scale

**Fig 1:**
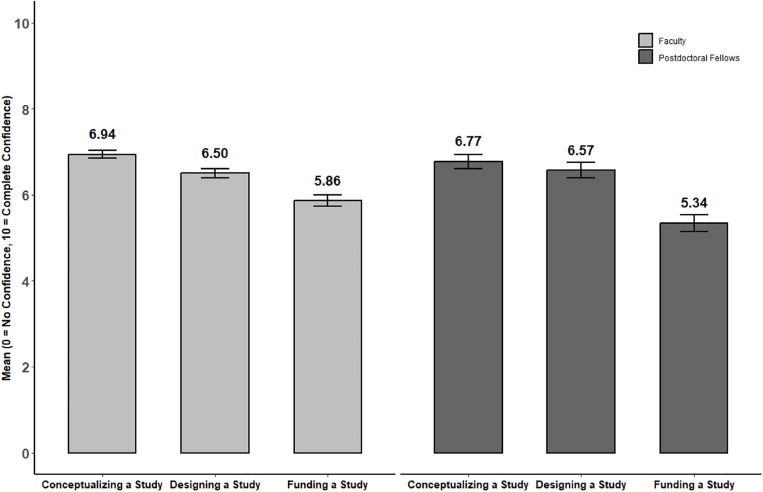
Baseline Scores for Grantsmanship Self-Efficacy. Confidence in ability to perform associated tasks; 19-item, 10-point response scale, from 0 = No confidence to 10 = Complete confidence. The mean scores for the subscales are calculated based on the number of items in each subscale: 8 items for “Conceptualizing a Study,” 7 items for “Designing a Study,” and 4 items for “Funding a Study.” The bars represent mean scores for self-efficacy in the three different aspects of grant writing. The error bars indicate the 95% confidence interval (CI) for each mean score, calculated as ±1.96 times the standard error (SE).

Baseline values for participants’ self-reported grantsmanship self-efficacy [33] are summarized in Fig 1. Scoring for each subscale is from 1 to 10 (1 = No confidence to 10 = Complete confidence). Mean subscale scores were similar for both faculty and postdoctoral fellows. Male faculty members reported significantly higher perceived self-efficacy for designing a study in comparison to female faculty: mean (SD) = 7.0 (1.6) versus 6.3 (1.9), p = 0.002. Otherwise, there were no significant differences by gender identity or URM status for either faculty or postdoctoral fellows. Of the 3 subscales, scores were lowest for self-efficacy in funding a study.

### Self-efficacy for career advancement scale

This 3-item scale [34,35] asks respondents to rate their level of agreement (1 = Strongly disagree, 2 = Disagree, 3 = Neither disagree nor agree, 4 = Agree, 5 = Strongly agree) for 3 statements: “*I feel advancement is as open to me as anyone else*,” “*I feel confident in my ability to progress in career*,” and “*I feel confident I can overcome any professional barriers*.” Item scores are summed then divided by 3 to produce a total score range of 1–5, with higher scores indicating higher levels of self-efficacy for career advancement. On this measure, the mean (SD) scores at baseline were 3.8 (0.7) for faculty and 3.8 (0.8) for postdoctoral fellows. Within each group, no significant differences were observed by gender identity or URM status.

### Key components of mentoring scale

This 6-item scale [36] asks respondents to indicate how much mentoring they are receiving at their institution (1 = None, 2 = A little, 3 = A moderate amount, 4 = A lot) to help them formulate career goals, plan how to achieve these goals, learn skills needed to succeed in their goals, find necessary resources, have a sponsor/champion to advance their career, and plan how to achieve their personal goals. Item scores are summed then divided by 6 to produce a total score range of 1–4, with higher scores indicating more mentoring for career advancement. On this measure, the mean (SD) scores at baseline were 2.4 (0.7) for faculty and 2.9 (0.7) for postdoctoral fellows. Once again, no significant differences were observed by gender identity or URM status for either group.

### Professional development support roles of current mentors

**Fig 2:**
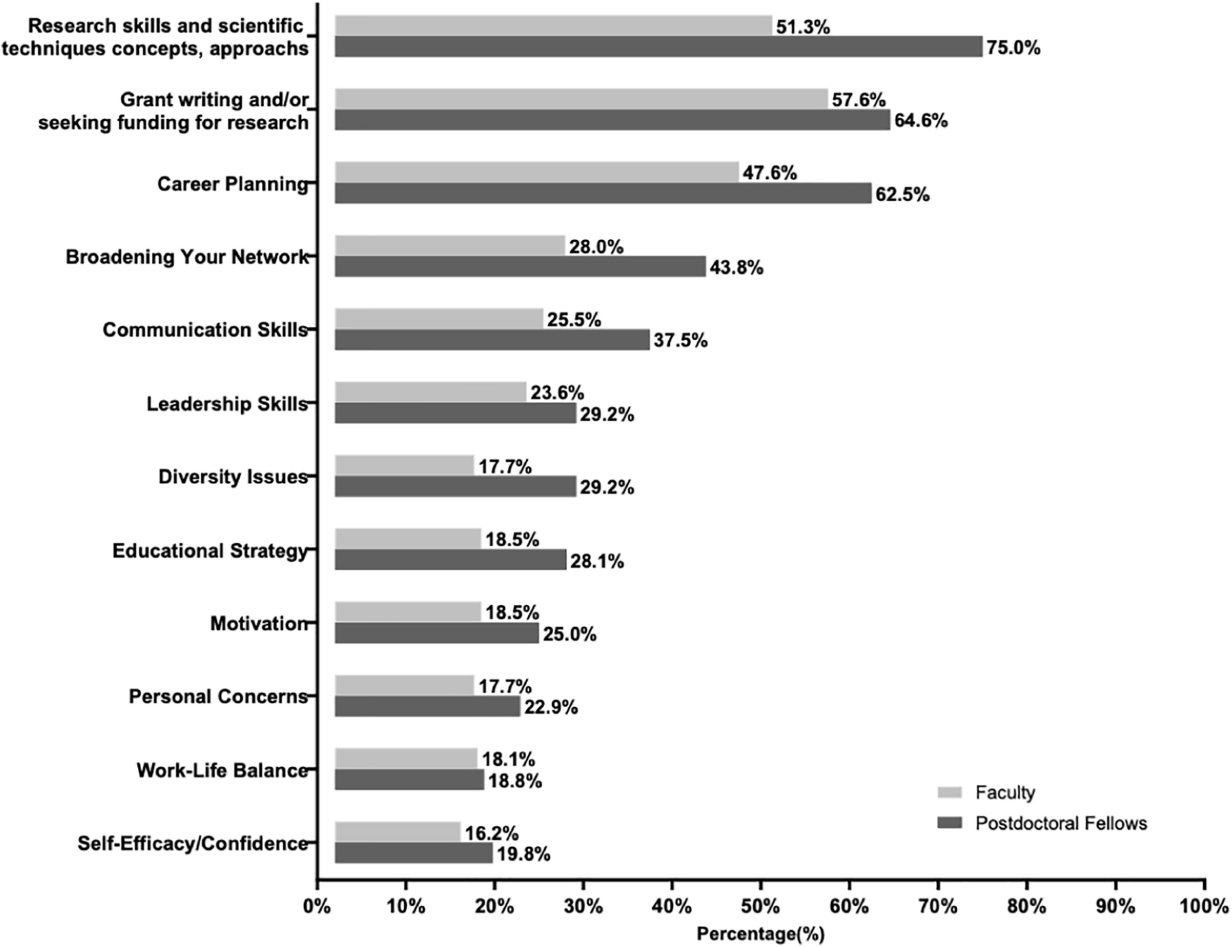
Discussion Topics in Mentoring Relationships. The figure illustrates the percentage of faculty and postdoctoral fellow respondents who selected “moderate” or “large extent” when asked how much they discuss specific topics with their mentors.

To acquire a general sense of the type of mentorship participants were receiving at baseline, they were asked, “*To what extent do you address and/or discuss with your mentors each of the following?”* The question was followed by 12 topics, with 5 response options each: 1 = Not at all, 2 = Small extent, 3 = Some extent, 4 = Moderate extent, 5 = Large extent. The percentage of respondents indicating either “moderate extent” or “large extent” for each item are graphed in Fig 2. Overall, the majority of faculty and postdoctoral fellows reported having access to mentors with whom they frequently discuss (indicated by “moderate” or “large extent”) 3 key aspects of their professional development as scientists: expansion of their research knowledge and skills, grant writing strategies, and career planning. Other mentorship topics were discussed at lower frequency. Across all topics, postdoctoral fellows indicated having more discussion with mentors than faculty, who may or may not have formal mentors. URM postdoctoral fellows and faculty were more likely than non-URM postdoctoral fellows and faculty to report at least moderate levels of discussion with their mentors about diversity issues; a greater extent of discussion about work-life balance/integration was reported by URM postdoctoral fellows and about self-efficacy/confidence by URM faculty than non-URM postdoctoral fellows and faculty (S1 Table). Otherwise, no significant differences were observed by gender identity or URM status, regardless of whether p-values were unadjusted or Bonferroni-adjusted.

## Discussion

Learning the complex skill of turning research ideas into fundable grant applications is a milestone in the career development of biomedical scientists seeking independent research careers. The baseline data presented here provide an informative snapshot of the socio-demographic characteristics and research backgrounds of early-career faculty and postdoctoral fellows who engaged in a study of 4 versions of an NIH-focused grant writing coaching intervention. Overall, we were successful in recruiting a large national sample into the trial. Participants were diverse in many respects (gender identity, racial and ethnic URM status, career stage, research focus, publication record, prior grant seeking history), although individuals with PhDs and from research-intensive institutions were over-represented. Generally, participants reported having good self-efficacy in grantsmanship and career advancement, and at least modest mentoring support at study entry. For most baseline variables we did not see significant differences by gender identity or by racial and ethnic URM status, with the number of prior peer-reviewed publications being a notable exception. We discuss our baseline findings in more detail below, with attention to how they may impact our future analyses of study outcomes and the generalizability of those results.

The study was promoted as a professional development opportunity for individuals actively engaged in NIH grant application writing, not just an overview of grantsmanship, with a strong emphasis on writing readiness and the expectation that participants will have at least initial application ideas and some early drafts. With this framing, the target enrollment goal was achieved through 6 cohorts over 3 years. We were also able to achieve a critical element of the study design, namely enrolling a gender- and racially diverse population of scientists (46.3% URM, 65.9% female). Similar to findings from the previous work [15] by our research team, the current study shows that grant writing coaching groups can be convened successfully across institutional boundaries and career levels. Further, participants were willing to commit to being part of a randomized trial with only a modest description of the differences in the arms of the study. Our study thus supports the feasibility of recruiting a large and diverse national sample, over multiple cohort cycles, into group coaching interventions with a randomized study design.

One important observation is that the baseline characteristics of male and female participants, and those from URM and non-URM backgrounds, were similar for most of the measures we examined, including prior success in submitting applications and obtaining grants. This absence of significant between-group differences at baseline offers some assurance that no socio-demographic subgroup could be characterized as entering the study at a significant disadvantage with respect to factors that might influence the intervention’s outcomes. Based on these findings, we anticipate that any study outcome differences by gender identity and/or URM status will not reflect differences in prior experiences with application submissions; nevertheless, we will adjust for prior experiences in the future analysis of outcomes due to its theoretical importance as a potential confounder.

One exception to the equivalency in baseline measures that we observed was for peer-reviewed publication counts, which were higher on average for male and non-URM participants. Certainly, the quantity (and quality) of a grant applicant’s publication history is heavily scrutinized by peer reviewers. This disparity in publication based on URM status is notable in the context of research by others in this area. Ginther et al (2018) studied the association of NIH R01 funding likelihood with the investigators’ academic rank, training, scholarly awards, and publications as reported in their NIH biographical sketches and bibliometrics [37]. They found that publication history significantly predicted the R01 funding gap between Black and White investigators. Given the significant degree of variability on this metric in our sample, it may be possible in our future analyses to determine whether number of publications and/or first authorship is associated with the probability of submitting an application and/or its funding.

The Grantsmanship Self-Efficacy Scale is a validated measure that we are assessing at multiple time points over the course of the study as a secondary outcome. Baseline mean subscale scores for our sample were virtually identical to those reported when this instrument was applied to a very similar group of faculty and postdoctoral fellows during our early work in NRMN [26,33]. Based on that previous work, we expect participants will demonstrate self-efficacy gains for all subscales after completing the coaching intervention and at subsequent follow-up at 18 and 24 months. Notably, the original validation of this scale found a statistically significant association between mean self-efficacy scores and grant submission timing. Specifically, for every 1-point increase in the mean score, the odds of submitting a grant 6 months post-training increased by 69% [33]. The current study offers an opportunity to further test and expand on this finding within a randomized trial design setting, once submission and funding data are available for analysis.

The other measures on which this article reports—Self-Efficacy for Career Advancement Scale, Key Components of Mentoring Scale, and Professional Development Support Roles of Current Mentors—were included in our study as common metrics collected across multiple U01 studies funded as part of the NRMN initiative. Although these data are not central to our study of intervention effectiveness, they do help to more fully characterize the sample at study entry, particularly participants’ perceptions of how well their current mentoring relationships are supporting their professional development. One key finding from our baseline data analyses is that, on average, both faculty and postdoctoral fellows reported having good self-efficacy (in grantsmanship and career advancement) and at least modest mentoring support at study entry, including mentorship for seeking research funding and grant writing (the focus of our intervention). Thus, it appears most participants were actively seeking out ways to complement the support provided by their existing mentors and home institutions, rather than filling voids. The construction and intent of our intervention is to complement what individuals receive from mentors with a systematic small group coaching experience that draws from well-established teaching and learning designs. Findings about good self-efficacy among study participants are consistent with the existing literature. For example, Botham and colleagues (2020) found that participants in a multi-week grant writing academy entered the program with a modest level of self-efficacy (6.2 on a 10-point scale) [38]. However, unlike in the current study, their participants came from a single institution and were not enrolled as research study participants, and the investigators did not use an experimental design.

Responses to surveys with scaled or numerical responses (like those for grantsmanship and career advancement self-efficacy, mentorship at home institutions, and topics discussed with mentors) can be affected by social desirability bias. However, any bias that might be present would be due to prior experiences rather participation in the study itself, since these data were gathered after study enrollment but before engagement in any intervention.

The sample reflects self-selected participants (i.e., they became aware of the study, decided to apply, felt sufficiently prepared to engage in writing and give feedback to others, met the study’s inclusion and exclusion criteria, and consented to abide by the study protocol). Thus, participants cannot be considered representative of any national population. Several characteristics of our sample provide guidance on the likely generalizability of our findings on intervention outcomes. Participants are conducting research across a broad spectrum of research topics, but all in research areas supported by NIH. Most are engaged in clinical and translational research (i.e., social/behavioral, clinical, epidemiological, and many of the basic science research topics). A majority (61.8%−70.0%) of our faculty participants had not previously submitted a K- or R-series application. Therefore, our study outcomes will be most generalizable to the population of faculty investigators who are new to this type of application writing, versus those who have written several NIH applications in the past. The same is true for postdoctoral fellows in our sample, who had little experience with applications writing beyond fellowship or administrative supplement submissions. However, participants with PhDs and from institutions with high or very high research resources are overrepresented in our sample; this may further limit the generalizability of our results in view of prior reports by NIH that funding rates for investigators vary by degree [39] and institution types [40].

Our data described here can be compared with other national samples of early-career faculty and postdoctoral scholars engaged in biomedical research. Specifically, an analysis of faculty and postdoctoral scholars supported by NIH’s Clinical and Translational Science Awards (CTSA) KL2 program showed that in fiscal year 2018, approximately 58% of KL2 scholars identified as female compared with 52% of scholars supported on other NIH institutional K awards [41]. In fiscal year 2020, 22% of KL2 scholars identified as belonging to a racial or ethnic group that is underrepresented in biomedical research, compared with 18% for other NIH institutional K awards in the same year [41]. In contrast, the sample recruited for our grant writing intervention trial included a higher percentage of women (65.9%) and individuals from underrepresented racial and minority groups (46.3%).

With respect to interest in and potential value of grant writing training, a survey of 547 KL2 scholars in 2021 demonstrated that 96% of these scholars had grant writing training available to them, and that 81% utilized this training [42]. Overall, 69% of the KL2 scholars indicated the grant writing training contributed to a “large extent” or “much” to their future career success, with a significant percentage of these scholars receiving additional NIH funding during and after their KL2 support [42]. Thus, the findings from our study in future publications should contribute to designing grant writing training and coaching that is most effective and efficient for helping early-career investigators acquire this critical skill.

## Supporting information

S1 Table Discussion Topics in Mentoring Relationships. Participant responses by gender identity and URM status to the question: “To what extent do you address and/or discuss with your mentors each of the following?”
(PDF)
