# Supplementary material for: A randomized trial of grant writing coaching groups: Baseline analysis of early-career scientists’ research background, demographics, and mentorship variables
Source: PLoS One. 2025 Oct 22;20(10):e0334039. doi: 10.1371/journal.pone.0334039 (PMC12543177; doi:10.1371/journal.pone.0334039)
Supplement: S1 Table — Participant responses by gender identity and URM status to the question: “To what extent do you address and/or discuss with your mentors each of the following?” (PDF) [file pone.0334039.s001.pdf]

Supporting Information 1, Table A. Faculty Responses by Gender Identity and URM Status to the Question: "To What Extent Do You Address and/or Discuss with Your Mentors Each of the Following?"

	Gender[a]				URM			
**Items, n (%)**	**Male N=98**	**Female N=169**	**Total N=267**	**p-valueb**	**Non-URM N=148**	**URM N=123**	**Total N=271**	**p-value[b]**
**Research skills and scientific techniques, concepts, approaches**				0.376				0.627
Not at all / Small Extent / Some Extent	51 (52.0)	78 (46.2)	129 (48.3)		70 (47.3)	62 (50.4)	132 (48.7)	
Moderate / Large Extent	47 (48.0)	91 (53.8)	138 (51.7)		78 (52.7)	61 (49.6)	139 (51.3)	
**Communication skills**				0.385				0.676
Not at all / Small Extent / Some Extent	70 (71.4)	129 (76.3)	199 (74.5)		112 (75.7)	90 (73.2)	202 (74.5)	
Moderate / Large Extent	28 (28.6)	40 (23.7)	68 (25.5)		36 (24.3)	33 (26.8)	69 (25.5)	
**Leadership skills**				0.455				0.667
Not at all / Small Extent / Some Extent	72 (73.5)	132 (78.1)	204 (76.4)		115 (77.7)	92 (74.8)	207 (76.4)	
Moderate / Large Extent	26 (26.5)	37 (21.9)	63 (23.6)		33 (22.3)	31 (25.2)	64 (23.6)	
**Broadening your network**				0.889				0.587
Not at all / Small Extent / Some Extent	70 (71.4)	122 (72.2)	192 (71.9)		104 (70.3)	91 (74.0)	195 (72.0)	
Moderate / Large Extent	28 (28.6)	47 (27.8)	75 (28.1)		44 (29.7)	32 (26.0)	76 (28.0)	
**Career planning and developing independence**				0.999				0.544
Not at all / Small Extent / Some Extent	52 (53.1)	89 (52.7)	141 (52.8)		75 (50.7)	67 (54.5)	142 (52.4)	
Moderate / Large Extent	46 (46.9)	80 (47.3)	126 (47.2)		73 (49.3)	56 (45.5)	129 (47.6)	
**Educational choices and strategies**				0.746				0.346
Not at all / Small Extent / Some Extent	81 (82.7)	136 (80.5)	217 (81.3)		124 (83.8)	97 (78.9)	221 (81.5)	
Moderate / Large Extent	17 (17.3)	33 (19.5)	50 (18.7)		24 (16.2)	26 (21.1)	50 (18.5)	
**Grant writing and/or seeking funding for research**				0.609				0.388
Not at all / Small Extent / Some Extent	44 (44.9)	70 (41.4)	114 (42.7)		59 (39.9)	56 (45.5)	115 (42.4)	
Moderate / Large Extent	54 (55.1)	99 (58.6)	153 (57.3)		89 (60.1)	67 (54.5)	156 (57.6)	
**Personal concerns**				0.252				0.750
Not at all / Small Extent / Some Extent	84 (85.7)	135 (79.9)	219 (82.0)		123 (83.1)	100 (81.3)	223 (82.3)	
Moderate / Large Extent	14 (14.3)	34 (20.1)	48 (18.0)		25 (16.9)	23 (18.7)	48 (17.7)	

**Work-life balance/integration**				0.251				0.635
Not at all / Small Extent / Some Extent	84 (85.7)	134 (79.3)	218 (81.6)		123 (83.1)	99 (80.5)	222 (81.9)	
Moderate / Large Extent	14 (14.3)	35 (20.7)	49 (18.4)		25 (16.9)	24 (19.5)	49 (18.1)	
**Diversity issues**				0.184				0.010
Not at all / Small Extent / Some Extent	85 (86.7)	135 (79.9)	220 (82.4)		130 (87.8)	93 (75.6)	223 (82.3)	
Moderate / Large Extent	13 (13.3)	34 (20.1)	47 (17.6)		18 (12.2)	30 (24.4)	48 (17.7)	
**Self-efficacy/confidence**				0.607				0.049
Not at all / Small Extent / Some Extent	84 (85.7)	140 (82.8)	224 (83.9)		130 (87.8)	97 (78.9)	227 (83.8)	
Moderate / Large Extent	14 (14.3)	29 (17.2)	43 (16.1)		18 (12.2)	26 (21.1)	44 (16.2)	
**Motivation**				0.999				0.059
Not at all / Small Extent / Some Extent	80 (81.6)	137 (81.1)	217 (81.3)		127 (85.8)	94 (76.4)	221 (81.5)	
Moderate / Large Extent	18 (18.4)	32 (18.9)	50 (18.7)		21 (14.2)	29 (23.6)	50 (18.5)	

[a]No analyses were performed for the small group of participants (1 postdoctoral fellow, 4 faculty) who selected "other" as gender identity
[b]Conducting Fisher's Exact Test

Supporting Information 1, Table B. Postdoctoral Fellow Responses by Gender Identity and URM Status to the Question: "To What Extent Do You Address and/or Discuss with Your Mentors Each of the Following?"

	Gender[a]				URM			
**Items, n (%)**	**Male N=22**	**Female N=73**	**Total N=95**	**p-value[b]**	**Non-URM N=49**	**URM N=47**	**Total N=96**	**p-value[b]**
**Research skills and scientific techniques, concepts, approaches**				0.576				0.999
Not at all / Small Extent / Some Extent	4 (18.2)	19 (26.0)	23 (24.2)		12 (24.5)	12 (25.5)	24 (25.0)	
Moderate / Large Extent	18 (81.8)	54 (74.0)	72 (75.8)		37 (75.5)	35 (74.5)	72 (75.0)	
**Communication skills**				0.804				0.206
Not at all / Small Extent / Some Extent	13 (59.1)	46 (63.0)	59 (62.1)		34 (69.4)	26 (55.3)	60 (62.5)	
Moderate / Large Extent	9 (40.9)	27 (37.0)	36 (37.9)		15 (30.6)	21 (44.7)	36 (37.5)	
**Leadership skills**				0.597				0.179
Not at all / Small Extent / Some Extent	17 (77.3)	51 (69.9)	68 (71.6)		38 (77.6)	30 (63.8)	68 (70.8)	
Moderate / Large Extent	5 (22.7)	22 (30.1)	27 (28.4)		11 (22.4)	17 (36.2)	28 (29.2)	

**Broadening your network**				0.624				0.099
Not at all / Small Extent / Some Extent	14 (63.6)	40 (54.8)	54 (56.8)		32 (65.3)	22 (46.8)	54 (56.2)	
Moderate / Large Extent	8 (36.4)	33 (45.2)	41 (43.2)		17 (34.7)	25 (53.2)	42 (43.8)	
**Career planning and developing independence**				0.215				0.060
Not at all / Small Extent / Some Extent	11 (50.0)	25 (34.2)	36 (37.9)		23 (46.9)	13 (27.7)	36 (37.5)	
Moderate / Large Extent	11 (50.0)	48 (65.8)	59 (62.1)		26 (53.1)	34 (72.3)	60 (62.5)	
**Educational choices and strategies**				0.597				0.822
Not at all / Small Extent / Some Extent	17 (77.3)	51 (69.9)	68 (71.6)		36 (73.5)	33 (70.2)	69 (71.9)	
Moderate / Large Extent	5 (22.7)	22 (30.1)	27 (28.4)		13 (26.5)	14 (29.8)	27 (28.1)	
**Grant writing and/or seeking funding for research**				0.307				0.833
Not at all / Small Extent / Some Extent	10 (45.5)	23 (31.5)	33 (34.7)		18 (36.7)	16 (34.0)	34 (35.4)	
Moderate / Large Extent	12 (54.5)	50 (68.5)	62 (65.3)		31 (63.3)	31 (66.0)	62 (64.6)	
**Personal concerns**				0.266				0.147
Not at all / Small Extent / Some Extent	19 (86.4)	54 (74.0)	73 (76.8)		41 (83.7)	33 (70.2)	74 (77.1)	
Moderate / Large Extent	3 (13.6)	19 (26.0)	22 (23.2)		8 (16.3)	14 (29.8)	22 (22.9)	
**Work-life balance/integration**				0.999				0.037
Not at all / Small Extent / Some Extent	18 (81.8)	59 (80.8)	77 (81.1)		44 (89.8)	34 (72.3)	78 (81.2)	
Moderate / Large Extent	4 (18.2)	14 (19.2)	18 (18.9)		5 (10.2)	13 (27.7)	18 (18.8)	
**Diversity issues**				0.107				0.001
Not at all / Small Extent / Some Extent	19 (86.4)	48 (65.8)	67 (70.5)		42 (85.7)	26 (55.3)	68 (70.8)	
Moderate / Large Extent	3 (13.6)	25 (34.2)	28 (29.5)		7 (14.3)	21 (44.7)	28 (29.2)	
**Self-efficacy/confidence**				0.224				0.205
Not at all / Small Extent / Some Extent	20 (90.9)	56 (76.7)	76 (80.0)		42 (85.7)	35 (74.5)	77 (80.2)	
Moderate / Large Extent	2 (9.1)	17 (23.3)	19 (20.0)		7 (14.3)	12 (25.5)	19 (19.8)	
**Motivation**				0.999				0.815
Not at all / Small Extent / Some Extent	17 (77.3)	54 (74.0)	71 (74.7)		36 (73.5)	36 (76.6)	72 (75.0)	
Moderate / Large Extent	5 (22.7)	19 (26.0)	24 (25.3)		13 (26.5)	11 (23.4)	24 (25.0)	

[a]No analyses were performed for the small group of participants (1 postdoctoral fellow, 4 faculty) who selected “other” as gender identity
[b]Conducting Fisher's Exact Test
